# Engineered TCR-T Cell Immunotherapy in Anticancer Precision Medicine: Pros and Cons

**DOI:** 10.3389/fimmu.2021.658753

**Published:** 2021-03-30

**Authors:** Qijie Zhao, Yu Jiang, Shixin Xiang, Parham Jabbarzadeh Kaboli, Jing Shen, Yueshui Zhao, Xu Wu, Fukuan Du, Mingxing Li, Chi Hin Cho, Jing Li, Qinglian Wen, Tao Liu, Tao Yi, Zhangang Xiao

**Affiliations:** ^1^ Laboratory of Molecular Pharmacology, Department of Pharmacology, School of Pharmacy, Southwest Medical University, Luzhou, China; ^2^ South Sichuan Institute of Translational Medicine, Luzhou, China; ^3^ Department of Pathophysiology, College of Basic Medical Science, Southwest Medical University, Luzhou, China; ^4^ Department of Oncology and Hematology, Hospital (T.C.M.) Affiliated to Southwest Medical University, Luzhou, China; ^5^ Department of Oncology, The Affiliated Hospital of Southwest Medical University, Luzhou, China; ^6^ Department of Oncology Rehabilitation, Shenzhen Luohu People’s Hospital, Shenzhen, China; ^7^ School of Chinese Medicine, Hong Kong Baptist University, Hong Kong, Hong Kong; ^8^ Department of Pharmacy, The Affiliated Hospital of Southwest Medical University, Luzhou, China

**Keywords:** ImmTAC, immunosuppression, immunotherapy, T-cell receptors, suicide genes

## Abstract

This review provides insight into the role of engineered T-cell receptors (TCRs) in immunotherapy. Novel approaches have been developed to boost anticancer immune system, including targeting new antigens, manufacturing new engineered or modified TCRs, and creating a safety switch for endo-suicide genes. In order to re-activate T cells against tumors, immune-mobilizing monoclonal TCRs against cancer (ImmTAC) have been developed as a novel class of manufactured molecules which are bispecific and recognize both cancer and T cells. The TCRs target special antigens such as NY-ESO-1, AHNAK^S2580F^ or ERBB2^H473Y^ to boost the efficacy of anticancer immunotherapy. The safety of genetically modified T cells is very important. Therefore, this review discusses pros and cons of different approaches, such as ImmTAC, Herpes simplex virus thymidine kinase (HSV-TK), and inducible caspase-9 in cancer immunotherapy. Clinical trials related to TCR-T cell therapy and monoclonal antibodies designed for overcoming immunosuppression, and recent advances made in understanding how TCRs are additionally examined. New approaches that can better detect antigens and drive an effective T cell response are discussed as well.

## Introduction

In 2018, GLOBOCAN reported 18.1 million new cancer cases and 9.6 million related deaths worldwide (1). Recent studies have examined the role of neo-antigens in promoting immunotherapy pointing to their promising potential to treat cancer and increase overall survival rates of patients (2). Antibodies which inhibit T-cell inhibitory receptor, programmed death 1 (PD-1), and target its ligand on tumor surface, PD-L1, in order to decrease T cell tolerance in the tumor microenvironment (TME), have recently been approved by US Food and Drug Administration (FDA) (3). Suppression of PD-1 and PD-L1 has achieved promising antitumor effects in metastatic bladder, pancreatic, ovarian, breast, gastric, and renal-cell cancers (4–9). However, more potent and targeted approaches are crucial to combat certain cancers resistant to conventional therapies (10).

**Figure 1:**
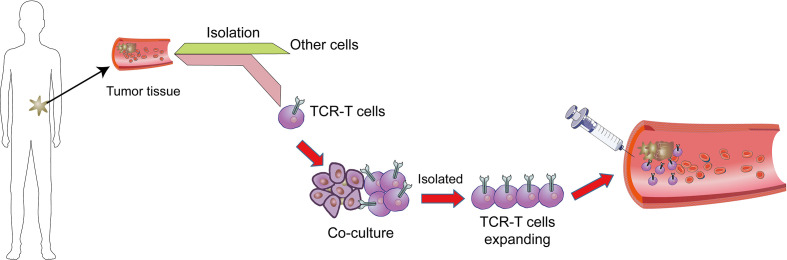
Adoptive T cell therapy. In adoptive cell therapy (ACT), cells are collected from cancer tissues/T cells which are then isolated from other cells, genetically manipulated by engineered TCR or CAR, co-cultured and proliferated, and eventually sent back into circulation.

The one of most powerful therapeutic strategy in immunotherapy is adoptive cell transfer (ACT) (**Figure 1**). Chimeric antigen receptors (CAR) and engineered T cell receptors (TCRs) are recent therapeutic manufactured receptors of T cells which are used in adoptive T-cell immunotherapy (11, 12). The TCR-engineered T cells express tumor antigen-specific receptors with α and β chains which are produced from high-quality and high-avidity antigen-specific T-cell clones. They are utilized to develop antigen-specific immunotherapy (13). Recent clinical trials assessing the effectiveness of ACT therapy, TCR modified T (TCR-T) cells, and immune checkpoint inhibitors showed good outcomes. The high affinity and cellular avidity of TCR-T cells determine specific binding of several cellular proteins, which mean TCRs play a key role in activating T cellular avidity. In contrast to low affinity of TCRs which reduce the efficiency of TCR-based therapy, the high-affinity TCRs (Affinity ≥ 2.5nM) are specific and sensitive for targeting cell-surface human leukocyte antigens (HLAs) (14). The TCR molecules belong to a superfamily of immunoglobulin and they consist of two covalently-bound polymorphic subunits, each of which is antigen-specific, and they are related to at least four different types of signal transduction chains.

**Figure 2:**
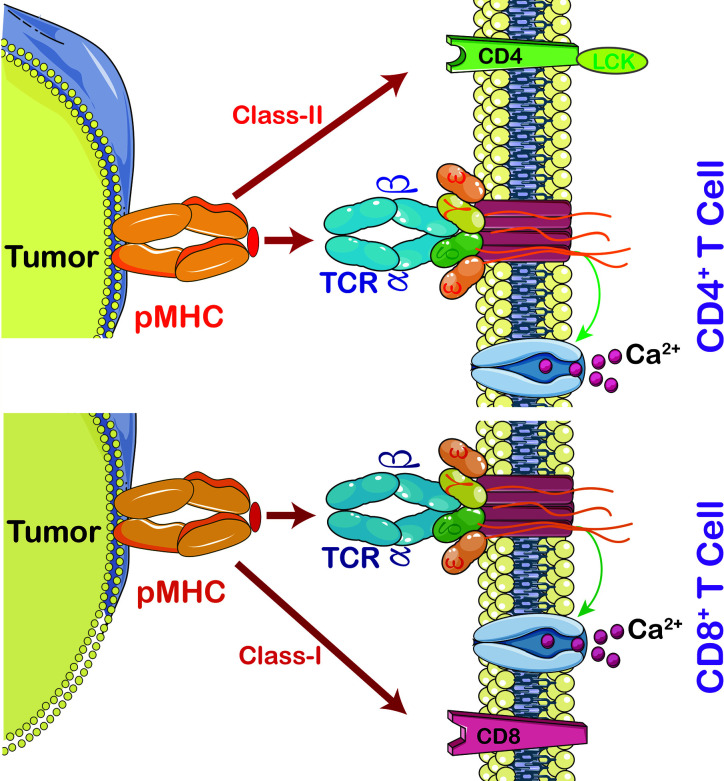
The interaction between peptide MHC and T cells. Cytotoxic T cells (CD8^+^) recognize MHC-I, CD3 coupled TCRs which activate T cell signaling cascade, including calcium-dependent and protein kinase C signaling pathways (PKC). Calcium is required for activation of nuclear factor of activated T cells (NFAT) leading to expression and secretion of Interleukin (IL)-2 and activation of PKC leading to tuning and regulating the production and secretion of ILs, including IL-2, T cell migration, T cell proliferation, and autoimmunity and graft rejection. CD4^+^ T cells are able to switch off the immune system by promoting regulatory T cells (Treg). In order to activate T cell proliferation, TCR also triggers p38MAPK and PI3K/Akt pathways. Once CD4 or CD8 activates TCR, it then activates Lymphocyte-Specific Protein-Tyrosine Kinase (Lck) which triggers phosphorylation of CD3 ζ chains.

In order to activate T lymphocyte, there has to be an interaction between TCR and major histocompatibility complex (MHC) (**Figure 2**). The strength of interaction between TCRs and pMHC (peptide-MHC) determines the fate of immature thymocytes, which is very important for the survival of naive T cells. Thus, TCR-T immunotherapy technique activates the host’s immune system through efficient interaction with MHC, especially class II molecules; the latter are specifically recognized by TCR-T cells and CAR-T cells (15). The TCR-T cells penetrate tumors but CAR-T cells are mainly distributed on the tumor periphery to access surface antigens. This makes TCR-T cells more efficient in cancer treatment (16).

This review provides insights into TCR-T cells with a special focus on its role in clinical anticancer immunotherapy. It also discusses advantages and disadvantages of different approaches in anticancer immunotherapy, including immune mobilizing monoclonal T-cell receptors against cancer (ImmTAC), inducible caspase-9, and Herpes simplex virus-thymidine kinase (HSV-TK) systems. Clinical trials on TCR-T cell therapy are discussed as well. Further, various strategies in anticancer immunotherapy with a special focus on TCR-T cell therapy are examined, highlighting efficacy and safety of each method in diagnostic and targeted cancer immunotherapy.

## Comparison of CAR and TCR Therapies

**Figure 3:**
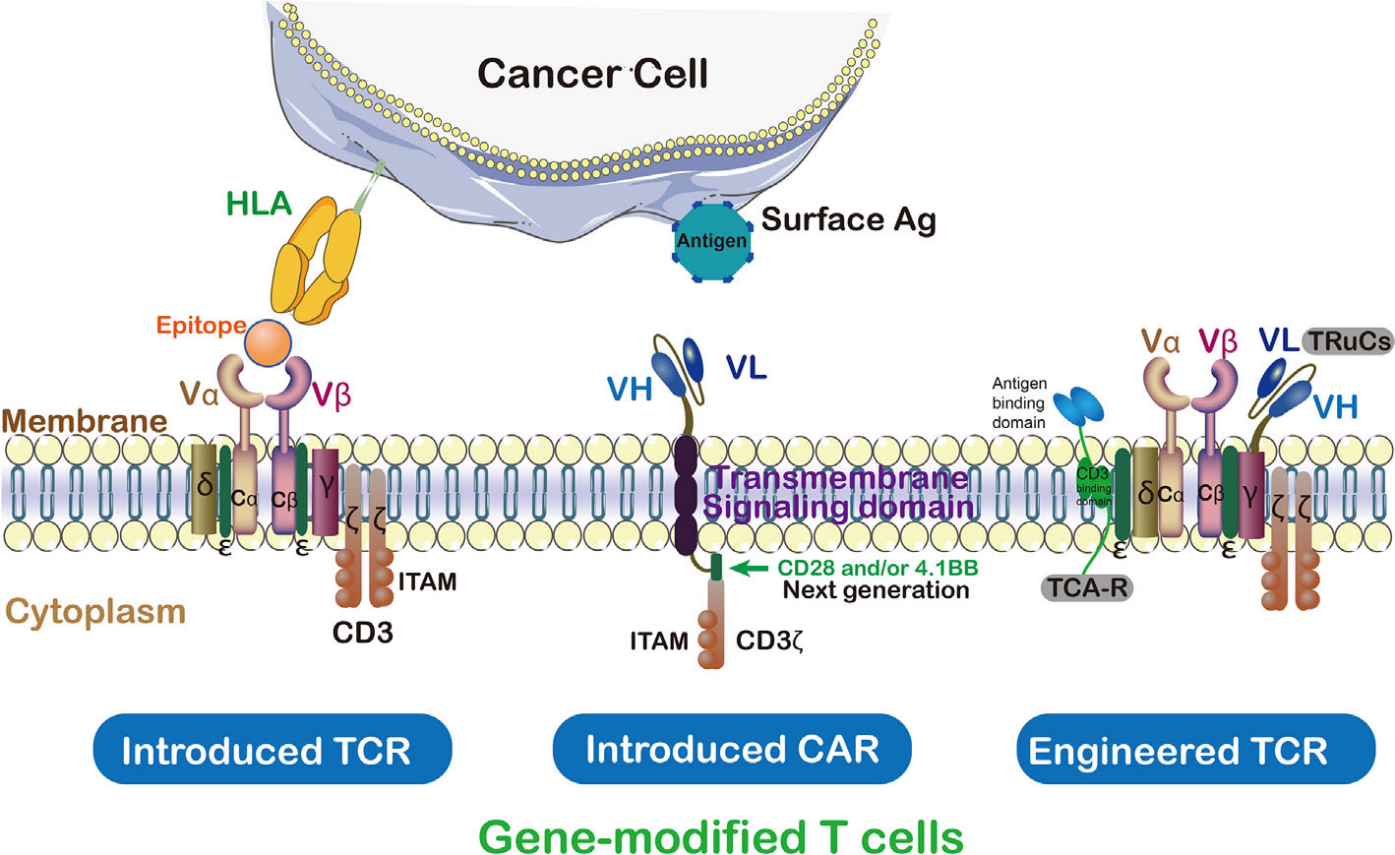
TCRs vs CAR and genetically modified T cells in immunotherapy. Cancer targeting receptors, TCR and CAR, are introduced to the activated T cells to empower them against special type of cancer. ζ subunit of CD3 on the surface of T cell is essential for triggering signaling cascade of T cells. In contrast to TCR which needs to be activated by endogenous CD3, CARs are hybrid receptors manufactured from a single-chain variable fragment (scFv) attached to ζ subunit of CD3. Both introduced and endogenous TCRs recognize HLA peptides on cancer cells. The CARs, instead, do not recognize HLA directly which trigger T cell signaling cascade in a TCR-independent manner.

In ACT, TCRs and CARs therapies, modified T cells have been successfully used as a paradigm-shifting clinical immunotherapy to treat solid tumors (17) (**Figure 3**). CAR T cells were engineered to transfer arbitrary specificity onto an immune effector cell, like T cell, which specifically eliminates antigen-bearing tumor cells (17). The CAR has scFv derived from antibody, CD3ζ and transmembrane domain (so-called first-generation CARs) (18). In this way, engineered CAR is able to recognize specific tumor associated-antigens (19). Therefore, the CAR has the ability to bind unprocessed tumor surface antigens without MHC processing (20) while TCRs engage with both tumor intracellular and surface antigenic peptides embedded in MHC (**Table 1**) (21). Generally, CAR scFv domain is used to engage with cell surface antigens (22). The scFv fragments could guide the constitutive activation and proliferation of T cells in an antigen-dependent mechanism; however, some scFv may guide T cells into an antigen-independent mechanism which lead to an unsuccessful treatment (23).

**Table 1:** Comparative characteristics of TCR and CAR.

Receptor Property	TCR	CAR
MHC involvement	Requires MHC matching	MHC-independent
Disadvantage	Redundant cytokine releasing	Off-tumor toxicities
Subunits	10	1
ITAMs number	10	3
Antigens required on target cells	1	100
Range of affinities for antigen	10^4^-10^6^/M^-1^	10^6^-10^9^/M^-1^
Recognition enhancing	Higher-order	Surface antigen density

Furthermore, first-generation CARs T cells showed limited expansion and relatively short persistence, which failed to evoke robust anti-tumor activity in the clinical studies (24, 25). The ‘second-generation’ CARs clinical activity can be induced by the CD3ζ domain insertion, namely co-stimulatory receptors CD28 (28ζ), 4-1BB/CD137 (BBζ) and OX40 (OX40ζ) CARs (26–28). The CD28 or CD137/4–1BB was added to CD3ζ endodomain of CAR-T cells which then promotes a more robust and durable T cells response (29, 30). Moreover, these second-generation CARs, which target CD19 antigen (CD19-specific scFv), are highly active against B cell malignancies and have promising clinical benefit (31, 32). In order to overcome the limitations of each individual costimulatory domain, the third generation CARs were proposed to simultaneously combine two co-stimulatory signaling (CD28 and 4-1BB), which presents a superior expansion and longer persistence than second-generation (33).

In contrast, TCRs are α/β heterodimers that bind to the MHC-bound antigens (**Figure 3**). As discussed above, CARs recognize tumor antigen which led to T cell activation with different functions compared with TCR. CAR-T cell therapy has certain disadvantages like off-tumor toxicities when targeting tumor-specific antigen (34). Compared with CARs, TCRs have several structural advantages in T cell-based therapy because they, such as more subunits in their receptor structure (ten subunits vs one subunit), greater immunoreceptor tyrosine-based activation motif (ITAMs) (ten vs three), less dependence on antigens (one vs 100), and more co-stimulate receptors (CD3, CD4, CD28, *etc.*) (20). The TCRs with low MHC interaction affinity range (10^4^-10^6^M^-1^) have been suggested for efficient T-cell stimulation (35). On the contrary, CARs possess a higher affinity range of (10^6^-10^9^M^-1^) and off-rates to recognize cell surface antigen (20). In order to maintain high-antigen sensitivity and to recognize pMHCs, exclusively monomeric TCR-CD3 complexes have been suggested (21). In contrast, the CAR-mediated cell sensitivity depends on higher density of cell surface antigen (36). Furthermore, T cell/antigen interaction is initiated in an immune synapse (IS) structure in which the TCR presents a ring region with peripheral LFA-1 adhesion, while CAR shows diffuse LFA-1 distribution without ring region (37). As a result, TCR-IS initiates a slower but longer duration signaling than that of CAR-IS. Meanwhile, the CAR-T cells presents faster killing function and move on to the next tumor target (serial killing), this was in stark contrast to the TCR-T cells protracted signaling and more extended killing.

Clinical trials involving with TCRs and CARs therapies have potential to suppress tumors progression. The CARs have been unable to effectively suppress malignant cells of some solid tumors and this is due to the presence of various types of antigens, their expression level, immunosuppressive environment, and construction of CARs. However, TCR-T cell transfer therapy has effectively treated some solid and hematological tumors (37).

## Recombinant TCRs

The TCR is one of the body’s most complex receptors, which contain six different receptor subunits to its very broad signaling activities in T cells (38). Tumor-infiltrating lymphocytes (TILs)-TCR changes dramatically influence the tumor-dominant T cells (39). Among which, the changes in TCR will contribute to the expansion/proliferation of T cells. The TCR diversity and increased TILs were associated with anti-tumor effects, and TCR engineering in selective TILs is optimal therapy for the tumor rather than periphery (40). The TCR is composed of α and β chains that together bind to the peptide-MHC ligand, and signaling subunits of CD3 complex (ϵ, γ and δ) as well as the CD3ζ homodimer (41). All subunits except CD3ζ have extracellular immunoglobulin (Ig) domains. Based on these structures, the immune mobilizing monoclonal modified T-cell receptors (ImmTAC) were designed so that can specifically recognize the target HLA-peptide. There are some emerging techniques that utilize signaling subunits of the TCR and improve immunotherapy efficiency in HLA-independent manners, such as ImmTAC, TRuCs and TAC.

### Immune Mobilizing Monoclonal T-Cell Receptors (ImmTAC)

**Figure 4:**
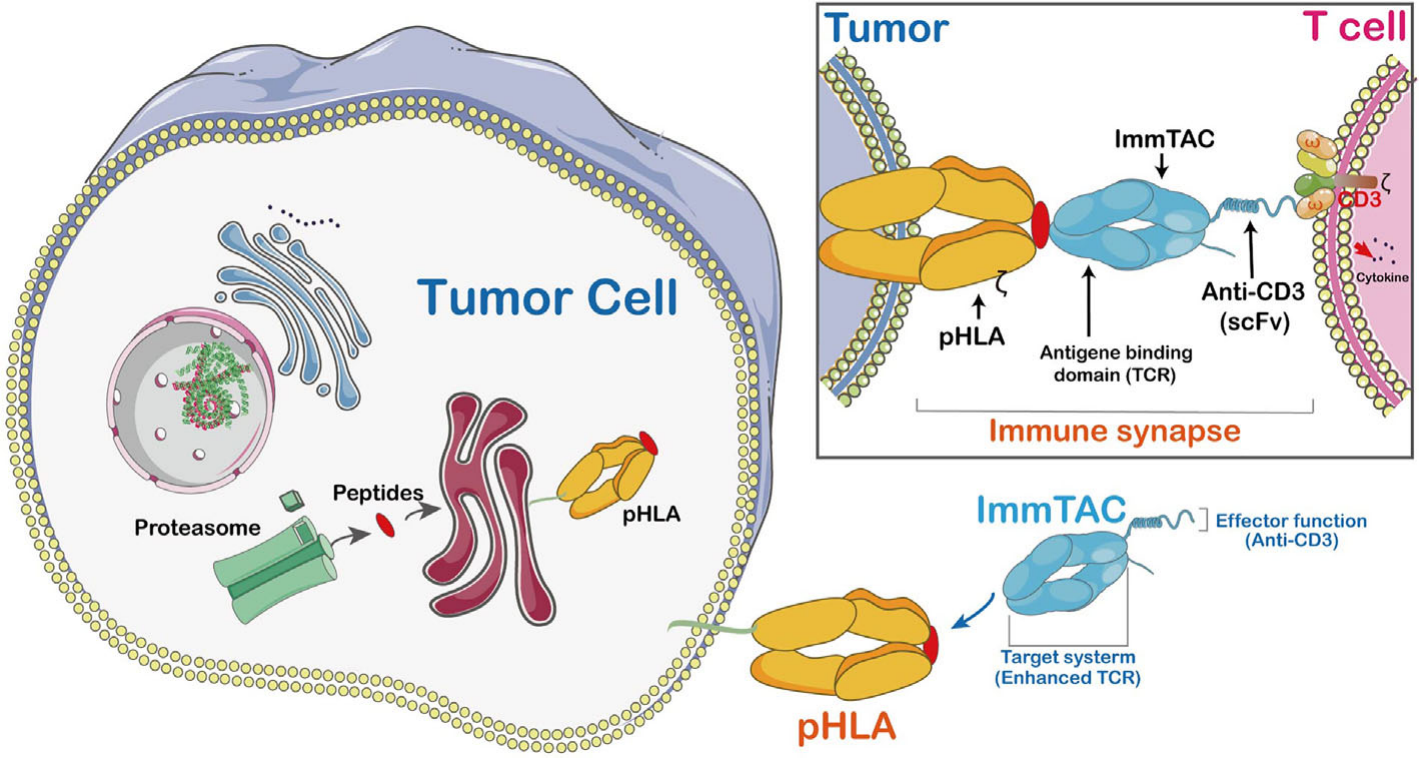
The mechanism of ImmTAC action. ImmTACs are designed to activate T cells against cancer and virus-infected cells. ImmTAC is specific to both MHC-peptides (pHLA) located on the surface of the tumor and CD3s located on T cells. The ImmTAC is able to directly activate CD3 and its corresponding pathways in activated T cells. In fact, the specific role of CD4 and CD8 is to stabilize the immune synapse, an important process of T cells shortened and empowered by ImmTACs.

The ImmTACs were designed using engineered, soluble, and affinity-enhanced monoclonal TCRs (mTCRs). ImmTACs are basically fusion proteins which combine an engineered TCR-based targeting system with a single chain antibody fragment (scFv) effector function. In the construction of ImmTACs, TCRs was deemed as antibodies which possess antigen recognition within the immune system. However, while antibodies only target cell surface or secreted proteins, TCRs are able to recognize peptides derived from intracellular targets presented by human leukocyte antigen (HLA) (42)(**Figure 4**).

ImmTACs have the potential to suppress tumor growth as they exert their activity through T cell redirection. Generally, ImmTACs comprising tumor-associated antigen-specific monoclonal TCR which strongly boost the affinity to pMHC (43). They effectively redirect these cells to eliminate cancer cells (44). The ImmTAC binds to the cancer cell through specific targeting of HLA-peptide complexes on their cell surface. The picomolar affinity of TCR to pMHC results in the coating of target cells by ImmTACs, and facilitates T-cell mediated effector function *via* interaction between scFv antibody fragments and CD3. Moreover, ImmTAC also activates CD8^+^ T cells in a dose-dependent manner with a low picomolar range of EC_50_ values (45). It has been shown that an ImmTAC, IMCgp100 effectively redirects and activates effector and memory CD8^+^ and CD4^+^ cells (46). The ImmTAC exhibits a polyfunctional response through secretion of several types of cytokines, such as tumor necrosis factor-α (TNF-α), interferon-γ, IL-6 pro-inflammatory cytokines, macrophage inflammatory protein-1α-β (MIP1α-β) and IFN-γ-inducible protein-10. The TNF-α and IFN-γ are highly effective inflammatory agents which promote apoptosis of tumor cell and stimulate inflammation of endothelial cells to enhance immune cell adhesion and extravasation in TME (47). MIP1α-β proteins involved in tumor proliferation or associated inflammation are potent chemo-attractants for monocytes. The MIP1α-β proteins are only secreted by CTLs and are mainly modulated by CD4^+^ T lymphocytes. An earlier study has shown IMCgp100-redirected T cells have a polyfunctional phenotype, a powerful anti-cancer response (46).

Furthermore, selecting appropriate target antigens is crucial for manufacturing ImmTACs. Certain dysregulated non-mutated proteins are presented as a tumor-associated antigen. Two decades ago, it was necessary to distinguish whether these targets are useful for immunotherapy by comparing their expression levels between tumor and normal tissues. Mass spectrometry has been used to identify low-abundance peptides because it has the sensitivity and ability to identify post-translational modifications for tumor antigens. While, failure to detect a pHLA complex using mass spectrometry does not mean it is absent. A pHLA target has the ability to induce an antigen-specific T cell response. The MHC-multimer technology can be used to profile T cell responses from immuno-stimulated patients. It also facilitates identification of suitable antigens (46). In order to compare ImmTAC targets, antigen expression, which is correlated with ImmTAC-mediated response, can be analyzed *in vitro* and *in vivo* studies (48). Of note, unexpected off-target reactivity of TCR-engineered T cells has been led to fatal cardiac toxicity, which was observed to recognize both epitope of MAGE-A3 and unrelated muscle protein titin in cardiac tissue in two series of clinical events (49). Hence, natural presentation of off-target peptide must be first confirmed using mass spectroscopy, and then followed by *in vitro* assessment of ImmTAC recognition. Since the therapeutic window maybe affected by ImmTAC off-target interactions, their affinity should be estimated (44). Overall, ImmTACs have been shown to enhance TCR-T cell anti-tumor response, but its safety needs further scrutiny.

### T Cell Receptor Fusion Constructs (TRuCs)

More recently, reprogrammed TCR-T cells with a new target specificity and the potential for HLA-independent cells was developed. T cell receptor fusion constructs (TRuCs), antibody-based binding domain fused to T cell receptor (TCR) subunit, which was designed for effective recognition of tumor surface antigens (29, 50). The TRuCs, consisted of specific ligand antibody fused to the extracellular N-termini of five TCR subunits (TCRα, TCRβ, CD3ϵ, CD3γ and CD3δ), provide the engineered T cell with new target specificity and HLA-independent target cell elimination ability which can be activated by corresponding target cells (51). Upon lentiviral transduction, the TRuCs will be integrated into native TCR complex on the T cells surface. Therefore, activation and effector function of T cells are retained. This method on TCR engineering showed better anti-tumor effect compared to the second-generation CAR-T cells. Moreover, the TRuCs dominate full signaling machinery of the TCR complex, while CARs only utilized limited signaling of isolated CD3ζ cytoplasmic tail (50).

### T Cell Antigen Coupler (TAC)

The T cell antigen coupler (TAC) is another platform that co-opts the endogenous TCR with MHC-independent manner to induce more efficient anti-tumor responses and reduces toxicity (52). The TAC chimeric proteins coupled the TCR to recognize antigen *via* CD3 domain binding, resulting in a TCR/CD3 complex formation and achieving more T cell responses (52). In addition, the activity of TAC receptor was critically dependent upon the choice of CD3-binding domain; hence, the appropriate scFvs improve the combination of phenotypic and functional characteristics. For example, the scFv derived from OKT3 (muromonab-CD3), one of the most commonly used agonistic anti-CD3 antibodies has lower cytokine production and cytotoxicity compared to UCHT1 (53). Thus, the delicate difference in integrating with the CD3 complex may contribute to substantially different functional outcomes. Compared to the second-generation CARs, the TAC engineered T cells did not merely favor the greater infiltration of solid tumors after adoptive injected but also reduced T cells expansion in healthy tissues that express antigens and off-tumor toxicities.

### Others

Recently, several novel ACT technologies have been proposed. The Natural killer cells (NKs) were emerged as a promising source of CAR-based therapies (CAR-NKs), which is safety and availability (54). The NKs initiate innate immune responses against infections and malignancies with natural cytotoxicity (55). The CAR modified NKs shown specific and potent cytotoxic activity against target cells (56), and the CAR-NKs treatment significantly reduced the tumor growth (57, 58). Some preclinical data indicated that CAR-NKs may be advantageous over CAR-T cells in T-lymphoid malignancies, as the shared expression of targetable antigens on both malignant and normal T lymphocytes (e.g. CD5 and CD28) (54, 59). Moreover, recent studies have demonstrated that stem cells can differentiate into functional immune cells and/or crosstalk with immune cells to modulate the tumor immune environment (60). The pluripotent stem cells differentiated Cytotoxic T cells and NKs shown several advantages compared to primary immune cells, such as improved anti‐tumor activity and produced in essentially unlimited numbers (61). Meanwhile, induced pluripotent stem cells (iPSC) as a novel cancer vaccine achieved a promising preventive and therapeutic effects to various types of cancers (62). Kooreman et, al. indicated that autologous iPSC-based vaccine can elicit anti-tumor responses in breast cancer, mesothelioma, and melanoma models and reduce the local or distant relapse after primary tumor resection (63). Among which, in melanoma, iPSC vaccine is associated with fewer Th17 cells and promoted antigen-specific anti-tumor T cells response. Furthermore, the iPSC plus CpG vaccine can evoked a strong cancer-immunity by upregulating mature APCs, effector T cells and cytotoxic T cells, as well as decreasing the amount of regulatory T cells (Tregs) (62).

## TCRs Side-Effects

The ACT with genetically engineered T cells has shown high sensitivity but with some severe adverse events in some clinical studies (11). Optimal TCR affinity in engineered T cells is vital and accordingly, receptor avidity is able to determine the safety/efficacy of T cell therapy (64). For example, in melanoma and neuroblastoma, some preclinical studies indicated the advanced intensity and durable antitumor effect of T cells. In a recent multi-target cell-based immunotherapy, patients were treated with CAR-Ts/TCRs against certain tumorigenic antigens, such as interventional studies of 10 and 4 tumor specific CAR-T/TCR in combination with cyclophosphamide or fludarabine (NCT03638206; NCT03941626). Meanwhile, the treatment-related adverse events were monitored based on Common Terminology Criteria for Adverse Events (CTCAE) v4.03 within 30 days after the last infusion. Not surprising, increased TCR expression and high frequency of TCR modified T cells within the graft improved TCR-modified T cells anti-tumor potency. In addition, combing the TCR therapy with irradiation therapy further improves efficiency of TCR therapy (65) (**Table 2**).

**Table 2:** Clinical trials activated on engineered TCR-T cells.

ID	Study Title	Cancers	Interventions	Participants
NCT03578406	HPV-E6-Specific Anti-PD1 TCR-T Cells in the Treatment of HPV-Positive NHSCC or Cervical Cancer	Cervical Cancer Head and Neck Squamous Cell Carcinoma	**Drug:** HPV E6-specific TCR-T cells	20
NCT03941626	Autologous CAR-T/TCR-T Cell Immunotherapy for Solid Malignancies	Esophagus Cancer Hepatoma Glioma Gastric Cancer	**Biological:** CAR-T/TCR-T cells immunotherapy	50
NCT03891706	Individualized Tumor Specific TCR- T Cells in the Treatment of Advanced Solid Tumors	Lung Cancer Melanoma	**Drug:** tumor-specific TCR-T cells	30
NCT03638206	Autologous CAR-T/TCR-T Cell Immunotherapy for Malignancies	B-cell Acute Lymphoblastic Leukemia Lymphoma Myeloid Leukemia (and 13 more…)	**Biological:** CAR-T cell immunotherapy Multi-target tumor specific CAR-Ts for CD19 and CD22 in B cell leukemia and lymphoma, CD33 in myeloid leukemia, B-cell maturation antigen (BCMA) and CD38 in multiple myeloma, NY-ESO-1 in multiple myeloma, esophagus cancer, lung cancer, and synovial sarcoma, DR5 in hepatoma, C-met in hepatoma, colorectal cancer, ovarian cancer and renal carcinoma, EGFR V III in hepatoma, lung cancer and glioma, and mesothelin in gastric cancer, pancreatic cancer and mesothelioma	73
NCT03139370	Safety and Efficacy of MAGE-A3/A6 T Cell Receptor Engineered T Cells (KITE-718) in HLA-DPB1*04:01 Positive Adults With Advanced Cancers	Solid Tumor	**Drug:** KITE-718 **Drug:** Cyclophosphamide **Drug:** Fludarabine **Device:** MAGE - A3/A6 Screening Test	75
NCT03691376	NY-ESO-1 TCR Engineered T Cell and HSC After Melphalan Conditioning Regimen in Treating Participants With Recurrent or Refractory Ovarian, Fallopian Tube, or Primary Peritoneal Cancer	HLA-A*0201 Positive Cells HLA-DP4 Positive Cells Platinum-Resistant Ovarian Carcinoma (and 6 more…)	**Biological:** Aldesleukin **Biological:** Autologous NY-ESO-1-specific CD8-positive T Lymphocytes **Other:** Cellular Therapy **Drug:** Melphalan	15
NCT02858310	E7 TCR T Cells for Human Papillomavirus-Associated Cancers	Papillomavirus Infections Cervical Intraepithelial Neoplasia Carcinoma *In Situ* (and 2 more…)	**Biological:** E7 TCR cells **Drug:** Aldesleukin **Drug:** Fludarabine **Drug:** Cyclophosphamide	180
NCT03686124	TCR-engineered T Cells in Solid Tumors	Refractory Cancer Recurrent Cancer Solid Tumor, Adult Cancer	**Biological:** IMA203 Product **Device:** IMADetect	16
NCT03029273	NY-ESO-1 TCR (TAEST16001)for Patients With Advanced NSCLC	Lung Cancer, Nonsmall Cell, Recurrent	**Drug:** Cyclophosphamide and Fludarabine **Biological:** Anti-NY-ESO-1 TCR transduced T cells	20
NCT03503968	TCR Modified T Cells MDG1011in High Risk Myeloid and Lymphoid Neoplasms	Safety Tolerability Feasibility Treatment Efficacy	**Drug:** MDG1011 **Other:** Investigator Choice therapy	92
NCT03912831	Safety and Efficacy of KITE-439 in HLA-A*02:01+ Adults With Relapsed/Refractory HPV16^+^ Cancers	Human Papillomavirus 16^+^ Relapsed/Refractory Cancer	**Drug:** KITE-439 **Drug:** Cyclophosphamide **Drug:** Fludarabine	75
NCT03247309	TCR-engineered T Cells in Solid Tumors With Emphasis on NSCLC and HNSCC (ACTengine)	Solid Tumor Cancer Head and Neck Squamous Cell Carcinoma Non-small Cell Lung Cancer	**Biological:** IMA201 Product **Diagnostic Test:** IMA_Detect **Diagnostic Test:** ACT-HLA	16
NCT03441100	TCR-engineered T Cells in Solid Tumors Including NSCLC and HCC Patients	Solid Tumor, Adult Cancer Hepatocellular Carcinoma (and 4 more…)	**Drug:** IMA202 Product **Device:** IMA_Detect	16
NCT02650986	Gene-Modified T Cells in Treating Patients With Locally Advanced or Stage IV Solid Tumors Expressing NY-ES0-1	Adult Solid Neoplasm	**Drug:** Cyclophosphamide **Other:** Laboratory Biomarker Analysis **Biological:** NY-ESO-1 Reactive TCR Retroviral Vector Transduced Autologous PBL **Biological:** TGFbDNRII-transduced Autologous Tumor Infiltrating Lymphocytes	24
NCT03431311	T Cell Receptor Based Therapy of Metastatic Colorectal Cancer	Colorectal Cancer	**Biological:** Adoptive Cell Therapy (ACT)	5
NCT03326921	HA-1 T TCR T Cell Immunotherapy for the Treating of Patients With Relapsed or Refractory Acute Leukemia After Donor Stem Cell Transplant	HLA-A*0201 HA-1 Positive Cells Present Juvenile Myelomonocytic Leukemia Recurrent Acute Biphenotypic Leukemia (and 24 more…)	**Biological:** CD8^+^ and CD4^+^ Donor Memory T-cells-expressing HA1-Specific TCR **Drug:** Fludarabine Phosphate **Other:** Laboratory Biomarker Analysis	24
NCT03462316	NY-ESO-1-specific T Cell Receptor (TCR) T Cell in Sarcoma	Bone Sarcoma Soft Tissue Sarcoma	**Biological:** NY-ESO-1(TCR Affinity Enhancing Specific T cell Therapy)	20
NCT02686372	TCR-Redirected T Cell Infusions to Prevent Hepatocellular Carcinoma Recurrence Post Liver Transplantation	Hepatocellular Carcinoma	**Biological:** HBV antigen specific TCR redirected T cell	10
NCT02719782	A Study of TCR-Redirected T Cell Infusion in Subject With Recurrent HBV-related HCC Post Liver Transplantation	Recurrent Hepatocellular Carcinoma	**Biological:** TCR-T	10

Interventions include Drugs, Medical devices, Biological agents and Other products that are either investigational or already available.

With on-target off-tumor toxicities, avidity becomes a main obstacle to the clinical success of ACT. When an antigen-specific receptor is used, in terms of efficacy, the avidity should be high enough for proper T cell activation (66). On the other hand, low avidity TCR interaction is sufficient to activate T cells, but strong avidity is required to sustain T-cell expansion (67). In phase I/II ACT clinical trial, the low-avidity engineered T cells showed safer profile, but they had a weaker anti-tumor response (68). Therefore, optimal avidity is a key factor in safety/efficacy of ACT. Recently, the reversible Ni^2+^-nitrilotriacetic acid histidine tags (NTAmers) technique was developed to efficiently separate high-avidity cytotoxic T-cells (69). Through recognizing TCR–pMHC interaction of T cells, engineered T cells can be isolated into high and low-intermediate avidity subtypes. Herein, an advance technology for avidity monitoring is necessary to ensure safe treatment.

### Herpes Simplex Virus Thymidine Kinase

Immunotherapy using T lymphocytes is an attractive strategy to treat many types of malignancies. However, the side-effects and off-target of T cell immunotherapy necessitates finding a safety switch mechanism which should be based on engineered T cells. Many clinical trials tried to eliminate potentially harmful cells using suicide genes (70). Thymidine kinase gene derived from herpes simplex virus I (HSV-TK) is one of the most common suicide genes. Therefore, transcriptional connection between HSV-TK and cell-division gene (CDK1) has been engineered and quantified based on mathematical models to determine the safety of this therapy (71). Cell batches in the suicide system and a homozygous HSV-TK-CDK1 boost safe-cell level (SCL) while ensuring a clinically validated safety range.

**Figure 5:**
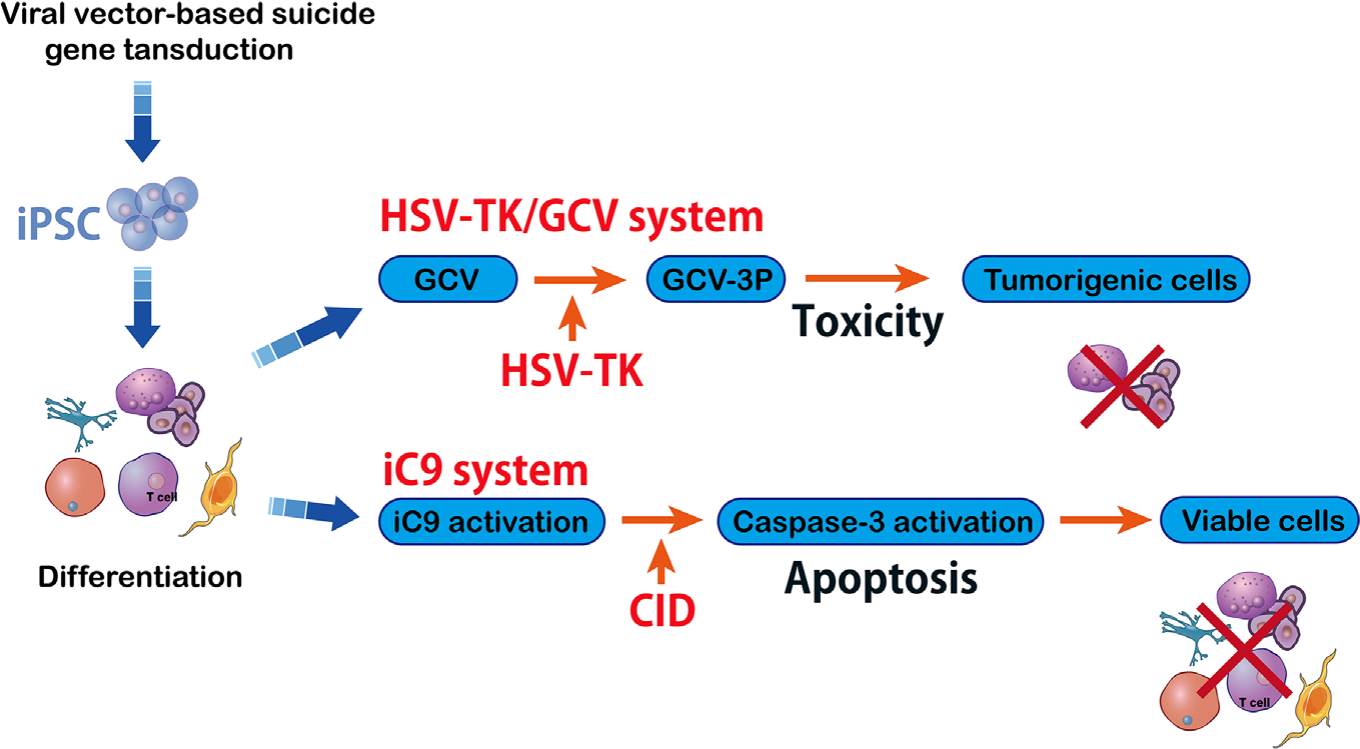
HSV-TK/GCV system vs inducible caspase-9 system. The herpes simplex virus–Thymidine Kinase/ganciclovir (HSV-TK/GCV) system eliminates tumorigenic cells and it is efficient and specific against inducible pluripotent stem cells (iPSC) that can kill the cells whose HSV-TK expression has been silenced. The HSV-TK/GCV system is used as a safety switch and it produces a toxic compound that kills the transduced cells. Another method in suicide gene therapy is introducing inducible caspase-9 (iC9) into iPSC. The iC9 dimerization activates iC9 which then triggers a caspase cascade leading to elimination of tumors originating from iPSC. Specific chemical inducers of dimerization (CID) induce iC9.

A serious complication related to hematopoietic allografts can happen when the Graft-versus-host disease (GvHD) is occurred. This, as a result of T cell transplantation, can cause tissue and organ damage. Transplantation of T cells recognizes the host’s histocompatibility in about 80% of patients (72). In order to prevent GvHD, T cells have been genetically modified with suicide gene HSV-TK using prodrug ganciclovir (GCV) (73)(**Figure 5**). Safety switches (suicide genes) are of particular value in long-term cell-dependent immunotherapies. They also avoid T cell off-target interactions. Herein, the suicide genes were able to control therapeutic process and can be initiated by early clinical interventions. For example, in clinical trial, HSV-TK modified T cells can be monitored by positron emission tomography (PET)/CT when they are migrating to an unexpected location (74).

There are several limitations. First, previous studies observed a small number of patients with HSV-TK engineered T cells had lost sensitivity to GCV. Second, the pro-drug GCV not only activates HSV-TK which precludes its administration, but it also acts as an anti-viral drug for other indications like cytomegalovirus (CMV) infections (75). Finally, HSV-TK engineered T cells have potential immunogenic activity. Autologous immune response to activate the suicide gene may result in transduced T-cell elimination, and therefore, reduces their therapeutic efficiency (76). Non-immunogenic suicide genes with low toxicity, stable expression, and high eliminating strength are urgently required in newly engineered transduced T cells.

### Inducible Caspase-9

Although HSV-TK shows safety in cell-based immunotherapy, the phosphorylated nucleoside analogs into DNA synthesis is required to complete elimination of tumor cells (77). Specifically, a quick elimination of the infused cells is required for cancer cellular therapies and regenerative medicine. An original inducible T-cell safety switch is brought to the donor T cell called caspase-9 (78) (**Figure 5**). Inducible caspase-9 is a fusion of human induced caspase-9 (iC9) which is a modified human FK-binding protein and it can be activated *via* a small-molecule compound AP1903 (79). This process depends on mitochondrial apoptotic pathway. After the pro-drug administration, the iC9-mediated cell clearance ratio was raised to ninety percent in half an hour (79). The iC9 suicide gene is less immunogenic, triggering reduced immune response against transgenic cells. This is to maintain a stable cell level in patients. After AP1903 treatment for one or two weeks in one study, polyclonal iC9-positive T cells were detected in peripheral blood with specific reactivity (79). Thus, the iC9 cell-suicide system is proven to be activated to guard T-cell-based immunotherapies and expand their clinical applications. The iC9-based safety switch has been shown to have better potential than the preexisting suicide genes for cellular therapy. In previous studies, iC9-T cells have been reported which deplete their alloreactive components *ex vivo (* 80). A new study showed iC9 allodepletion could be done *in vivo* instead, and iC9-T cells can be eliminated within 30 minutes of AP1903 absorption (81).

The transferred T cells can also secrete pro-inflammatory cytokines leading to life-threatening GvHD-associated cytokine release syndrome (CRS) (82). The CRS is caused by increased levels of cytokines including IL-6 and IFN-γ. Immunosuppressants, for example tocilizumab as an anti-IL-6 receptor, with or without corticosteroids, can reverse this situation (83). The results indicate iC9 activation is sufficiently potent to promote allodepletion and treat GvHD, which leads to rapid resolution of CRS. Despite the iC9 system is engineered to prevent side-effect of T cell immunotherapy, integration of any transgene is mutagenic and potentially oncogenic. Hence, it is vital to assess its potential risks and benefits.

## Discussion and Concluding Remarks

Tyrosine kinases belong to a key category of oncogenic proteins including Her2, EGFR, and VEGFR which are targeted by tyrosine kinase inhibitors (TKIs), such as lapatinib, gefitinib and sunitinib (84, 85). However, treatment with TKIs showed acquired resistance to chemotherapy after a few weeks of administration (86). Hence, lower toxicity and higher efficacy in anticancer therapeutic strategy are vital. Recently, gene-modified T cells with least adverse effects have been shown as extremely effective in treating solid and liquid tumors. Therapies which activate the immune system, such as those using antibodies against immune checkpoint PD-1, were shown to have a great potential (87). In addition, antigen-targeted approaches of monoclonal antibodies, CAR-T cell therapy, and TCR-based therapy have shown varied successes against specific tumors (11, 88). Therapies that use modified TCR-T cells for preclinical and clinical investigations have tremendous potential (89, 90). In lung cancer, however, solo use of ImmTAC therapy has failed. In order to recovery ImmTAC potency and tumor regression, the anti-PD-1 monoclonal antibody showed beneficial effects (91). ImmTAC efficiently redirects and activates effector and memory T cells within the CD8^+^ and CD4^+^ repertoires. This results in cytotoxic activity against melanoma cells. Apart from its direct therapy effect, ImmTAC can redirect lymphocytes to secrete several key cytokines and chemokines in T cells, such as IFN-γ, ILs (IL-2 and IL-6) and TNF-α, which result in a sustained anti-tumor defense in ACT (46).

The TME is known to suppress TILs (92). Engineered TCRs are able to boost T-cell trafficking and activation in TME (93, 94). Recently, E7 TCR gene therapy showed potential therapeutic value against HPV cancers (89). The ACT from TCR engineered human T cells has shown encouraging results. However, these approaches need further validation (95). In ACT, GVHD maybe observable with different and unpredictable kinetics (96). It has also been shown TCR/CD3 inhibition triggers T cell apoptosis (97).

A quick elimination of infused cells is important for cancer cellular therapies as engineered T cells expressing suicide genes with a specific prodrug are rapidly eliminated by apoptosis. Caspase-9 is a key element in apoptosis and is involved in various chemotherapy stimuli (78). Incorporating NK cells with an iC9 suicide switch enhances safety of anti-leukemia approaches which allows for their safe clinical application (98). However, this method also leads to a rapid inactivation of effector T cells in the event of adverse reactions (99). Anti-cancer and suicide mechanisms can co-exist without effects on cells and hamper tumoricidal activity (100). The iC9 safety switch of transduced T-cells also proved to be reliable in Phase I trial involving patients undergoing haplo-identical stem cell transplantation (90). Furthermore, HSV-TK has been observed to be another safety switch to trigger apoptosis in the cell line of head and neck carcinoma (101). Novel and highly efficient technique called TransfeX delivers HSV-TK into cervical, oral and pharyngeal with tumoricidal effects (102). Hence, TCR-T cell engineered with HSV-TK suicide gene may also enhance oncotherapy. It is evident therefore that TCR-T-cell immunotherapy has the potential to eradicate tumors in addition to lowering risks associated with immunotherapy (103).

In conclusion, TCR-T ACT has a good potential to treat cancers. TCR tuning is vital for T cell re-activity, immune responses, and its clinical effects on foreign antigens. The engineered TCRs targeting special antigens also boost efficiency of immunotherapy. The safety of genetically modified T cells for ACT is also vital. The present article reviewed advances of TCR T cell immunotherapy and proximal new techniques which can detect antigens and drive a T cell response. However, there are some limitations which need to be addressed in future research.

## Author Contributions

QJZ, YJ, JS, PJK and SXX wrote the paper, YSZ, XW, FKD and MXL edited the manuscript, CHO, JL and QLW prepared and adjusted the figures, TL, TY and ZGX designed the study, provided funding and reviewed the manuscript. All authors contributed to the article and approved the submitted version.

## Funding

This work was supported by the National Natural Science Foundation of China (Grants: 81503093, 81972643, and 81672444) and the Joint Funds of the Southwest Medical University & Luzhou (Grants: 2016LZXNYD-T01, 2017LZXNYD-Z05 and 2019LXXNYKD-07) and Science & Technology Department of Sichuan Province(Grants: 2018JY0079).

## Conflict of Interest

The authors declare that the research was conducted in the absence of any commercial or financial relationships that could be construed as a potential conflict of interest.
